# Identification of Ramie Genes in Response to *Pratylenchus coffeae* Infection Challenge by Digital Gene Expression Analysis

**DOI:** 10.3390/ijms160921989

**Published:** 2015-09-11

**Authors:** Yongting Yu, Liangbin Zeng, Zhun Yan, Touming Liu, Kai Sun, Taotao Zhu, Aiguo Zhu

**Affiliations:** Institute of Bast Fiber Crops, Chinese Academy of Agricultural Sciences, Changsha 410205, China; E-Mails: yuyongting@caas.cn (Y.Y.); zengliangbin@caas.cn (L.Z.); yanzhuntoronto@gmail.com (Z.Y.); liutouming@caas.cn (T.L.); sunkai@caas.cn (K.S.); zhtt1988@126.com (T.Z.)

**Keywords:** *Boehmeria nivea* (L.) Gaudich, root lesion nematode, digital gene expression, resistance related genes, cystatin

## Abstract

Root lesion disease, caused by *Pratylenchus coffeae*, seriously impairs the growth and yield of ramie, an important natural fiber crop. The ramie defense mechanism against *P. coffeae* infection is poorly understood, which hinders efforts to improve resistance via breeding programs. In this study, the transcriptome of the resistant ramie cultivar Qingdaye was characterized using Illumina sequence technology. About 46.3 million clean pair end (PE) reads were generated and assembled into 40,826 unigenes with a mean length of 830 bp. Digital gene expression (DGE) analysis was performed on both the control roots (CK) and *P. coffeae*-challenged roots (CH), and the differentially expressed genes (DEGs) were identified. Approximately 10.16 and 8.07 million cDNA reads in the CK and CH cDNA libraries were sequenced, respectively. A total of 137 genes exhibited different transcript abundances between the two libraries. Among them, the expressions of 117 and 20 DEGs were up- and down-regulated in *P. coffeae*-challenged ramie, respectively. The expression patterns of 15 candidate genes determined by qRT-PCR confirmed the results of DGE analysis. Time-course expression profiles of eight defense-related genes in susceptible and resistant ramie cultivars were different after *P. coffeae* inoculation. The differential expression of protease inhibitors, pathogenesis-related proteins (PRs), and transcription factors in resistant and susceptible ramie during *P. coffeae* infection indicated that cystatin likely plays an important role in nematode resistance.

## 1. Introduction

High-throughput sequencing (also called “Next-generation” sequencing) technology, such as Illumina (Solexa) sequencing, 454 pyrosequencing, and ABI SOLiD sequencing, allows us to analyze the transcriptome and whole genome-wide expression (digital gene expression, DGE) profiles of an organism or organ in a particular state, even in the absence of a reference genome. Additionally, it produces more comprehensive information and is faster than other available technology. It is also more cost-effective than genome sequencing. Thus, these technologies have quickly been accepted and are now widely used to explore differentially expressed genes (DEGs) when the organism or organ is in a particular growth or differentiation stage, or under abiotic or biotic stresses [1,2,3,4,5]. For instance, Zhang *et al.* used DGE to study maize resistance to *Sporisorium reilianum* f. sp. *Zeae* by comparing transcriptional changes in the roots of resistant and susceptible cultivar after pathogen infection [1].

Ramie (*Boehmeria nivea* (L.) Gaudich), also known by the common name “China grass”, is a perennial herbaceous plant in the nettle family Urticaceae. It is an important natural fiber crop primarily cultivated in China, India, and other Southeast Asian and Pacific Rim countries [2]. Aside from its production of high-quality natural fiber, ramie leaves and shoots are a good source of cow and goose feed. In addition, as perennial plant with complex roots system, ramie is effective in supporting water and soil conservation in south-east China [6].

Root lesion disease (RLD), a destructive root disease that is caused by the nematode *Pratylenchus coffeae*, severely impairs the growth and yield of ramie [7]. As the use of some chemicals, such as methyl bromide and aldicarb, is banned because of their negative environmental impact, few pesticides that are both effective and environmentally friendly are available for nematode control. In addition, because ramie is a perennial plant usually grown for 5–8 years, rotation with non-host crops is not a suitable method of controlling RLD. Thus, the cultivation of resistant cultivars is the preferred method of decreasing the damage caused by RLD. However, over the years, ramie breeders have paid much more attention to the improvement of yield and quality traits, rather than resistance. So far, little information about ramie RLD resistance mechanisms or genes is available. This severely hinders efforts in effectively breeding and using nematode-resistance ramie. More recently, the transcriptome changes of the ramie cultivar Zhongzhu 1 (a susceptible cultivar) in response to *P. coffeae* infection were studied [8]. The above-ground biomass (stems and leaves) was used, and the differential gene expression induced by nematode infection was studied; however, though it is the part of the plant most relevant to plant−nematode interactions, the root was not studied.

Very few RLN-resistance genes (besides *Rlnn1*, a gene originating from Excalibur wheat and located on chromosome 7ALz) have been identified, and the mechanism behind RLN-resistance is still not known [7,9]. Meanwhile, many dominant genes imparting resistance to other parasitic nematodes have been identified in plants, such as *Mi-1*, *Mi-3*, *Mi-9*, *hero*, *N*, *Hsipro-1*, *rkn1*, *rhg1* to *rgh4*, *Cre1* to *Cre7*, *CaMi*, *Gpa2*, *Rma*, and *Mex-1* [10,11,12,13,14]. In addition, some of these have been successfully utilized in transgenic plants [12]. Additionally, since the first reports detailing the transcription analysis of giant cells induced by root-knot nematode infection [15] and the differential expression of genes during the cyst nematode (CN) infection of susceptible soybean plants [16] were reported, increasing numbers of researchers have attempted to explore new resistance genes, virulence genes, and mechanisms relevant to responses to plant-RKN or -CN infection by studying differential mRNA expression. By comparing the transcriptome profiles of compatible and incompatible interactions between tomato plants and RKN, a gene encoding glycosyltransferase was found to be significantly regulated by nematode infection, and necessary for RKN resistance [17]. Recently, the whole transcriptome profiles of *P. coffeae* [18] and *P. thornei* [19] have been reported, which will facilitate the exploration of parasitism genes and host-pathogen interactions.

In this study, we constructed a transcriptome library for healthy roots, and two digital gene expression libraries corresponding to nematode-affected and unaffected resistant ramie cultivar roots, respectively. Based on these data, the ramie genes that are differentially expressed during interactions with *P. coffeae* were identified, and a potential resistance mechanism was discussed.

## 2. Results

### 2.1. De Novo Transcriptome Assembly

As the ramie genome has not been sequenced, it was necessary to acquire its transcriptome, as reference to use in the identification of differential gene expression induced by *P. coffeae*. The transcriptome of the control ramie roots (CK) was obtained using the Illumina sequencing platform. After filtering low quality sequences, about 46,305,614 clean pair end (PE) reads (4,676,454,795 base pairs) were obtained from a 200 bp insert library. Of these clean reads, 95.08% have Q20 bases (base quality greater than 20).

Using Trinity software, these reads were first assembled into 1,940,406 contigs with an average length of 72 bp, then into 73,531 transcripts with an average length of 1038 bp, and finally into 40,826 unigenes with an average length of 830 bp (Table 1). None of these unigenes were less than 100 bp, and 11,263 of them were no less than 1 Kb in size. The length distributions of the unigenes are showed in Figure S1.

**Table 1:** Summary of *de novo* sequence assembly.

Fragments Length	Total Number		
	Contigs	Transcripts	Unigenes
0–300	1,908,616	15,757	12,877
300–500	12,017	14,229	9821
500–1000	7806	15,142	6865
1000–2000	7608	18,196	7302
2000+	4359	10,207	3961
Total number	1,940,406	73,531	40,826
Total length	140,000,159	76,345,971	33,878,532
N50	99	1665	1491
Mean length	72	1038	830

### 2.2. Functional Annotation and Classification

In order to acquire comprehensive functional information, the 40,826 unigenes were searched against the National Center for Biotechnology Information (NCBI) non-redundant (Nr, http://blast.ncbi.nlm.nih.gov/), Swiss-Prot (http://web.expasy.org/docs/), the clusters of orthologous group (COG, https://www.ncbi.nlm.nih.gov/COG/), and the kyoto encyclopedia of genes and genomes (KEGG, http://www.genome.jp/kegg/) databases. Protein function information can be predicted using the annotation of the most similar proteins recorded in the databases. The searches revealed that 26,685 (65.36%), 18,895 (46.28%), 9657 (23.65%), and 6243 (15.29%) unigenes had homologous sequences in the Nr, Swiss-Prot, COG, and KEGG databases, respectively. In total, 26,861 (65.79%) unigenes were functionally annotated (Table 2). Another 13,965 (34.21%) unigenes exhibited no homology with the known sequences in the four databases.

**Table 2:** Summary of unigene annotation using different databases.

Annotated Database	Annotated Number	300 ≤ Length < 1000	Length ≥ 1000
Nr	26,685	10,427	11,092
SwissProt	18,851	7019	8632
COG	9657	2991	5132
KEGG	6243	2092	2843
Total annotated	26,851	10,510	11,105

**Figure 1:**
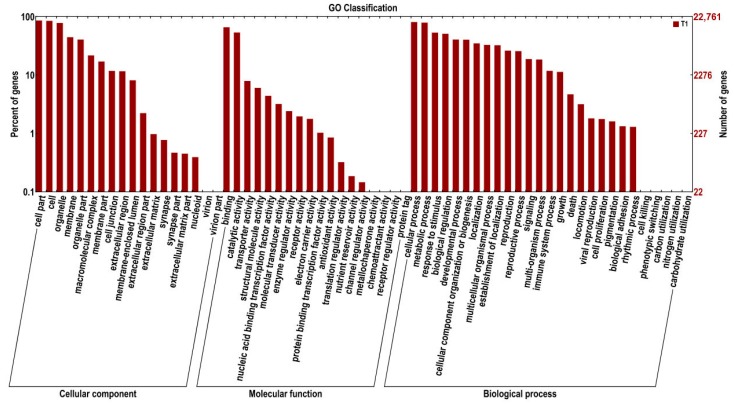
Gene Ontology (GO) classification of ramie roots transcriptome. The results are summarized in three categories: cellular component, molecular function and biological process. The y-axis on the right show the number of genes in category, and that on the left show the percentage of a specific category of genes in that main category.

Gene Ontology (GO) is an international standardized gene function classification system. Based on the Nr annotation information, we obtained the GO functional annotations of 20,286 out of the 40,826 total unigenes searched using the Blast2Go software program. Then, using the WEGO software program, these genes were categorized into 57 functional groups in three major categories: molecular functions, biological processes, and cellular components (Figure 1). As some genes matched to homologous proteins in the Nr database had more than one GO term, 20,286 unigenes were assigned to GO classes with 135,678 functional terms in total. Of these, assignments to the biological processes made up the largest portion (58,758, 43.31%), followed by cellular components (52,858, 38.96%), and molecular functions (24,062, 17.73%).

Clusters of Orthologous Group is a database in which orthologous gene products are classified. After searching the COG database, 9657 unigenes were annotated to 25 COG categories. Some genes were assigned to several COG categories, which lead to the use of 25 categories to include 13,369 unigenes. Among these categories, the “general function prediction only” category represents the largest group (2478, 25.66%), followed by “translation, ribosomal structure and biogenesis” (1507, 15.61%), and “replication, recombination and repair” (1144, 11.85%). The categories “cell motility” (11, 0%), “nuclear structure” (1, 0%), and “extracellular structures” (0, 0%), were the smallest groups of unigenes (Figure S2).

To identify the biological pathways active in ramie roots, we mapped the annotated sequences to the reference canonical pathways in the KEGG databases, the result of which showed that 6243 of the total unigenes were assigned to 116 pathways. However, since some of the genes were assigned to several KEGG pathways, the 116 pathways included a total of 6742 unigenes. Of these pathways, metabolic pathways represented the largest portion (2040 unigenes, 30.26%), defense pathways represented the second largest portion (969 unigenes, 14.37%), and 132 unigenes were involved in the “plant-pathogen interaction” pathway (Table S1).

### 2.3. Digital Gene Expression (DGE) Library Sequencing and Mapping to the Reference Transcriptome

DGE analysis was performed to identify genes which are involved in responses to *P. coffeae* attack. Using Illumina Hiseq2000 sequencing technology, approximately 10.16 and 8.07 million clean reads were obtained from CK and CH ramie roots libraries, respectively.

Matching efficiency refers to the proportion of reads in DGE library with matches to the reference sequences in transcriptome libraries obtained above. Via matching efficiency analysis, we can check if the data in the DGE library is normal, and if the quantification of gene expression is correct. Both of these affect the accuracy of the results of subsequent gene differential expression analyses. The matching efficiency of each DGE library against the transcriptome reference gene library (containing 40,826 unigenes) was examined; the mapped reads in the CK and CH libraries were 80.81% (with 99.27% identity) and 80.42% (with 99.24% identity) (Table S2), respectively, which indicated that the results were reliable. To detect if there were any nematode RNA in CH samples, the unmapped 1,580,260 reads (19.58%) were then mapped to reference sequences from *P. coffeae*. There were 1257 reads (0.02%) overlapped with 122 nematode transcripts sequences, indicated there were probably some gene orthologues from nematode in CH samples.

Sequencing saturation analysis of the two libraries showed that when the reads reached 5 M in the CK library and 4 M in CH library, respectively, no new genes were detected (Figure S3). Therefore, these two libraries were sequenced to saturation, leading to the complete representation of the transcripts produced under the tested conditions.

### 2.4. Identification of Differentially Expressed Genes (DEGs)

Gene expression information was obtained by mapping the clean reads to the transcriptome reference database, which contains the 40,826 distinct sequences obtained above. According to the normalized gene expression level, significantly differentially-expressed genes (at least a two-fold change) were identified in *P. coffeae*-challenged ramie roots (Figure S4). In total, 137 unigenes were markedly influenced by *P. coffeae* attack, with 117 and 20 of them being up- and down-regulated, respectively (Table S3). To detect if any DEGs were from nematode, sequences of 137 DEGs were compared with sequences in a local database constructed for *P. coffeae* using BLASTn. The result showed 6 DEGs shared sequence similarities with unigene of *P. coffeae*, indicating they were likely orthologues from nematode. These 6 DEGs were removed from DEG pool of ramie.

Of these DEGs, 32 (23.4%) were potential defense-related genes, as identified by functional annotation. These 32 genes were divided into 7 groups: protease inhibitors (PI) (6 unigenes), transcription factors (TFs) (4 unigenes), heat shock proteins (HSP) (4 unigenes), PR proteins (3 unigenes), antioxidant enzymes (2 unigenes), cell wall reinforcement (5 unigenes), and others (8 unigenes). Interestingly, all PI genes (Unigene19135: trypsin inhibitor, Unigene11292: cysteine protease inhibitor, also called cystatin, Unigene2589 and Unigene9323: protease inhibitor, Unigene13433: endogenous α-amylase/subtilisin inhibitor, and Unigene16750: xyloglucanase inhibitor 3), HSP genes (Unigene3204 and Unigene3206: heat shock protein 70, Unigene2183: 17.9 kDa class II heat shock protein, and Unigene11009: 23.6 kDa heat shock protein), 3 ethylene-responsive transcription factors (ERF) genes (Unigene9042, Unigene9043, and Unigene 17588), and 2 antioxidant enzyme genes (Unigene13732: polyphenol oxidase (PPO) and Unigene7762: superoxide dismutase (SOD)) were all upregulated (Table 3). Additionally, the functions of 33 (24.1%) DEGS were unclear because the predicted proteins were unknown and 24 (17.5%) DEGs showed no match in either the Nr or SwissProt database. The functions of five genes (Unigene4169, Unigene5613, Unigene7059, Unigene26831 and Unigene37351) (Table S3) expressed only in the *P. coffeae-*challenged roots were also unclear.

**Table 3:** Defense-related differentially expressed genes (DEGs) in *P. coffeae*-challenged ramie roots.

Gene ID	CH	CK	log_2_(CH/CK)	Regulated	Gene Annotation
**Protease Inhibitor**					
Unigene11292	271	134	1.02	Up	Cysteine protease inhibitor
Unigene2589	41	7	2.55	Up	Protease inhibitor
Unigene9323	38	3	3.66	Up	Protease inhibitor
Unigene19135	1589	504	1.66	Up	Trypsin inhibitor
Unigene13433	468	217	1.11	Up	Endogenous α-amylase/subtilisin inhibitor
Unigene16750	73	22	1.73	Up	Xyloglucanase inhibitor 3
**Heat Shock Protein**					
Unigene2183	25	2	3.64	Up	17.9 kDa class II heat shock protein
Unigene11009	61	14	2.12	Up	23.6 kDa heat shock protein
Unigene3206	29	3	3.27	Up	Heat shock protein 70
Unigene3204	23	2	3.52	Up	Heat shock70 kDa protein
**Transcription Factor**					
Unigene17588	88	26	1.76	Up	Ethylene-responsive transcription factor
Unigene9042	148	12	3.62	Up	Ethylene-responsive transcription factor
Unigene9043	131	10	3.71	Up	Ethylene-responsive transcription factor
Unigene12273	116	258	−1.15	Down	WRKY transcription factor
**Antioxidant Enzyme**					
Unigene13732	48	7	2.78	Up	Polyphenol oxidase
Unigene7762	359	168	1.10	Up	Superoxide dismutase
**Cell Wall Reinforcement**					
Unigene24997	19	1	4.25	Up	Arabinogalactan protein 23
Unigene24306	36	3	3.58	Up	Cell wall-associated hydrolase
Unigene9085	258	104	1.31	Up	Proline-rich cell wall protein
Unigene5928	24	2	3.58	Up	Cytochrome P450
Unigene14594	−60	127	−1.08	Down	Cellulose synthase-like protein
**Pathogenesis-Related Protein**					
Unigene24094	4572	1738	1.40	Up	Chitinase
Unigene10467	157	50	1.65	Up	Endochitinase
Unigene13343	−53	111	−1.07	Down	Non-specific lipid-transfer protein-like protein
**Others**					
Unigene47	21	1	4.39	Up	E3 ubiquitin protein ligase
Unigene20652	81	29	1.48	Up	E3 ubiquitin-protein ligase
Unigene15546	84	37	1.18	Up	F-box/kelch-repeat protein
Unigene23894	240	109	1.14	Up	Nitrate reductase
Unigene23899	90	42	1.10	Up	Nitrate reductase
Unigene8357	1008	369	1.45	Up	Non-symbiotic hemoglobin
Unigene10901	253	60	2.08	Up	Universal stress protein A-like protein
Unigene22463	−55	131	−1.25	Down	Lipoxygenase

### 2.5. Gene Expression Patterns Analysis Using qRT-PCR

**Figure 2:**
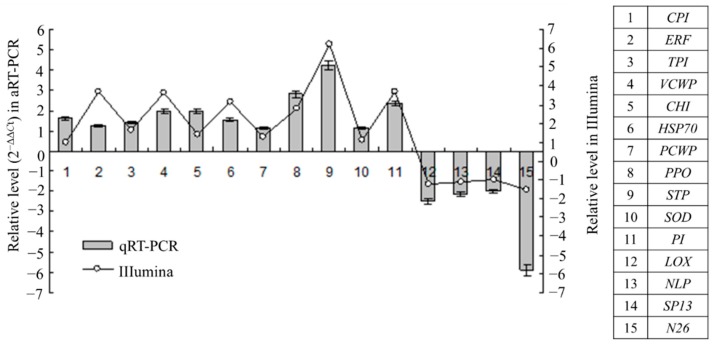
Validation of Illumina DGE results using quantitative real-time PCR (qRT-PCR) method. Data from qRT-PCR are means of three replicates and bars represent standerd error. *CPI*, cysteine protease inhibitor (Unigene11292); *ERF*, ethylene-responsive transcription factor (Unigene9043); *TPI*, trypsin inhibitor (Unigene19135); *VCWP*, vegetative cell wall protein (Unigene2183); *CHI*, chitinase (Uigene24094); *HSP70*, heat shock protein 70 (Unigene3206); *PCWP*, proline-rich cell wall protein (Unigene9085); *PPO*, polyphenoloxidase (Unigene13732); *STP*, ser/thr-rich protein T10 (Unigene5469); *SOD*, superoxide dismutase (Unigene7762); *PI*, proteinase inhibitor (Unigen9323); *LOX*, lipoxygenase (Unigene22463); *nsLTP*, non-specific lipid-transfer protein (Unigene13343); *SP13*, scarecrow-like protein 13-like (Unigene21351); *N26*, Nod26-like protein (Unigene6416).

To confirm the results of the digital gene expression analysis, qRT-PCR was also performed to analyze the expression patterns of 15 candidate genes using the equally-mixed sample containing the RNA from all time points. The results showed that 11 genes were upregulated and 4 genes were downregulated. This trend in the change in gene expression was in accordance with that detected via Illumina sequencing, which indicates that the results of the DGE analysis are credible (Figure 2).

**Figure 3:**
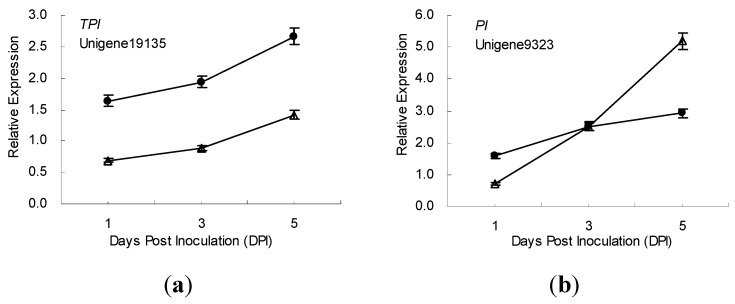

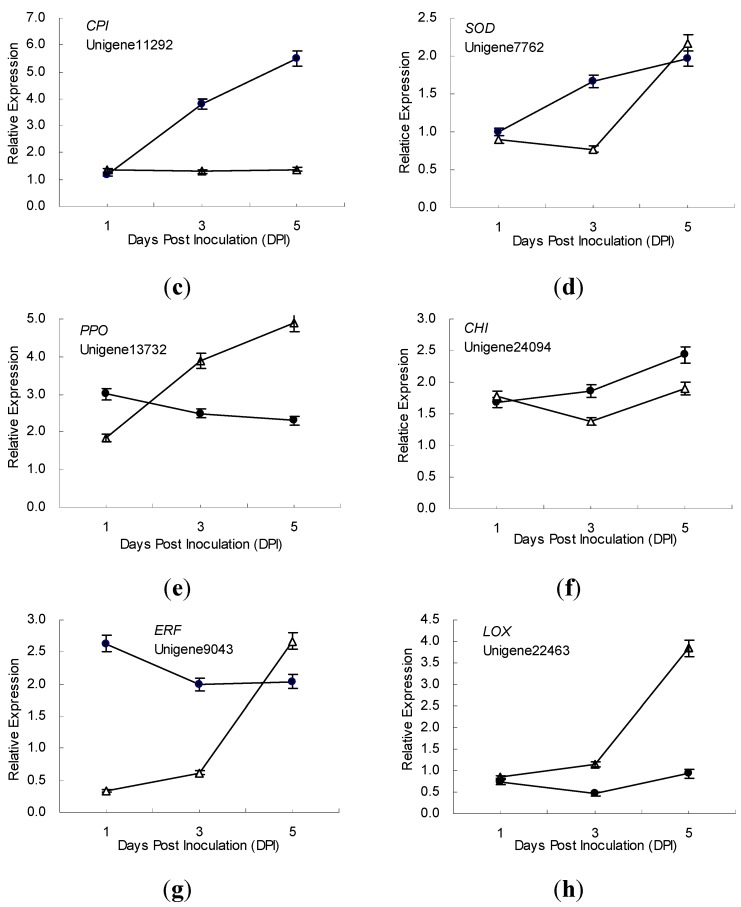
Time-course expression profiles of 8 defence-related genes in ramie roots during incompatible (●) and compatible (Δ) interactions with *P. coffeae* revealed by qRT-PCR analyses. (**a**) *TI*, Trypsin inhibitor; (**b**) *PI*, Proteinase inhibitor; (**c**) *CPI*, Cysteine protease inhibitor; (**d**) *SOD*, Superoxide dismutase; (**e**) *PPO*, Polyphenoloxidase; (**f**) *CHI*, Chitinase; (**g**) *ERF*, Ethylene-responsive transcription factor; and (**h**) *LOX*, Lipoxygenase. Data from qRT-PCR are means of three biological replicates (each has 2 experimental replicates) and bars represent standerd error.

The time-course expression profiling of 10 defense-related genes in the incompatible and compatible interactions between ramie and *P. coffeae* were also studied using samples from all 3 time points (1, 3 and 5 dpi) (Figure 3). During the incompatible interaction, the expression of 7 genes respectively encoding cystatin, ERF, trypsin inhibitor, chitinase, PPO, SOD, and a proteinase inhibitor (encoded by Unigene9323) were upregulated, and that of one gene (lipoxygenase, LOX) was suppressed (relative expression levels < 1) after *P. coffeae* challenge. Additionally, of the 7 upregulated genes, 5 genes exhibited increased expression; while 2 genes (*PPO* and *ERF*) exhibited decreased expression 1–5 days post inoculation (dpi).

Meanwhile, in the compatible interaction, four genes respectively encoding trypsin inhibitor, protease inhibitor (encoded by Unigene9323), SOD and chitinase exhibited expression profiles similar to those found in the incompatible interaction. The mRNA of the trypsin inhibitor increased from 1 to 5 dpi, but the expression level was lower than that of resistant ramie. The expression level of *PI* (Unigene9323) was lower than that in resistant ramie at 1 to 3 dpi, but higher at 5 dpi. The mRNA of *CPI* showed no obvious change in susceptible ramie after *P. coffeae* inoculation, but increased continuously in resistant ramie. One day after *P. coffeae* inoculation, the expression of LOX was slightly suppressed in susceptible ramie, similar to that in resistant ramie; however, it was upregulated in susceptible ramie at 3 to 5 dpi. The mRNA of *PPO* and *ERF* (Unigene9043) increased between 1 to 5 days after *P. coffeae* inoculation, exhibiting an expression pattern opposite expression that seen in resistant ramie (Figure 3).

## 3. Discussion

The molecular response of a host plant to *P. coffeae* has rarely been studied, and the underlying resistance mechanisms have so far been poorly understood. *P. coffeae*-induced DGEs in banana roots were isolated via suppression subtractive hybridization. Differential gene expression in resistant and susceptible cultivars revealed that the expression of defense genes increased faster in a resistant cultivar than in a susceptible one, and that the resistant cultivar initiated a response to the attack much more quickly than the susceptible cultivar [20]. More recently, Zhu *et al.* investigated the transcriptome changes in ramie (only the over-ground parts) due to attack by *P. coffeae*, using Illumina sequencing, and identified 777 DEGs [8]. They did not use a resistant cultivar or study DEGs occurring in the roots—the part of the plant first contacted by root pathogens—in response to nematode attack. Thus, the results cannot accurately explain ramie resistance to nematode attack. The time-course infection of ramie by *P. coffeae* has not been studied; however, it is known that *P. coffeae* and other *Pratylenchus* species can successfully penetrate the susceptible host roots within 5 days of inoculation [21,22,23]. Based on these finding, samples treated for 1, 3, and 5 days were used to study the early molecular response of ramie to *P. coffeae* challenge.

During the early stage of root challenge by *P. coffeae*, 137 DEGs of ramie were induced, and DGE analysis revealed most of them (85.4%) to be upregulated. Additionally, the expression profiles of 15 genes revealed by qRT-PCR agreed with the results of the DGE analysis, indicating there was a low rate of false DEGs present in our results. The DEGs obtained in this study were far fewer than the 777 DEGs identified by Zhu *et al.*, who used a susceptible ramie cultivar treated for 7 days, and analyzed gene expression via comparative transcriptome sequencing [8]. These material and experimental differences can be expected to produce different results. The expression profiles characterized by this study will add to our understanding of ramie resistance to *P. coffeae*.

During the early part of the incompatible interactions, PIs such as cystatin, trypsin inhibitors, protease inhibitors, and endogenous α-amylase/subtilisin inhibitors exhibited upregulated expression, but pathogenesis-related proteins (PRs) such as PR-1, β-1,3-glucanase, and peroxidase were not fund to be induced. In comparison, cystain, a protease inhibitor, and an endogenous α-amylase/subtilisin inhibitor were not regulated, and PR-1, β-1,3-glucanase, and peroxidase were induced in another susceptible ramie by *P. coffeae* [8]. During the incompatible and compatible interactions of plants in the *Musa* genus with *P. coffeae*, chintinase, β-1,3-glucanase, and peroxidase were regulated in both resistant and susceptible cultivars, but the expression levels in resistant cultivars were higher than those in susceptible cultivars [20].

Previous studies showed that cystatin (also called cysteine protease inhibitor, CPI) play important roles in the arms race between plant and nematode. Rice cystatin (Oc-I, Oc-II and Oc-IΔD86) confers increased resistance in transgenic tomato plants [24], *Arabidopsis thaliana* [25,26], alfalfa [27], banana [28], sweet potato [29], and lily [30] to nematodes such as *Globodera pallida*, Heterodera schachtii, *Meloidogyne incognita*, *Rotylenchulus reniformis*, *Pratylenchus penetrans*, and *Radopholus similis*. Additionally, taro cystatin (CeCPI) and maize cystatin (CCII) also exhibited anti-nematode activities in transgenic research [31,32]. After *P. coffeae* inoculation, the expression of genes encoding cystatin (Unigene11292), a trypsin inhibitor (Unigene19135), an endogenous α-amylase/subtilisin inhibitor (Unigene13433), and two protease inhibitors (Unigene2589 and Unigene9323) increased in resistant ramie roots. Meanwhile in the susceptible ramie, the expression of Unigene19135 increased, while Unigene11292 showed no obvious change in expression. From these results, we think that ramie cystatin likely plays an important role in nematode resistance.

Jasmonic acid (JA) can positively regulate the expression of PIs and nematode resistance [33,34,35]. In higher plants, JA and its derivatives are synthesized via the octadecanoid pathway, and LOX is key enzyme which oxidizes α-linolenic acid in the first step of this pathway [36]. In this study, the expression of a gene (Unigene22463) encoding LOX was suppressed in resistant ramie, while being upregulated in susceptible ramie after *P. coffeae* challenge. It seemed that the accumulation of cystatin in resistant and susceptible ramie was negatively related to that of LOX. Previous studies showed that maize 9-lipoxygenase (*ZmLOX3*) can enhance resistance to nematode attack, but not some fungi diseases [37,38], and that LOX-mediated fungal pathogen resistance is pathogen-specific [39]. Recent studies showed that two LOX isoforms (*LOX3* and *LOX4*) of Arabidopsis play opposite roles in resistance to nematode attack [40]. Therefore, the role of ramie LOX encoded by Unigene22463 is not clear, and requires further study.

Although transcription factors (TFs) usually act down-stream of the JA/ET signaling pathway and activate the transcription of defense-related genes [41,42], TFs such as WRKY and ERF can affect JA-mediated plant pest resistance [43,44,45]. Two WRKY transcription factors regulate the expression of JA biosynthesis genes (*LOX*, *AOS*, *AOC* and *OPR*), thereby increasing the levels of JA and JA-isoleucine produced [45]. In *P. coffeae*-challenged ramie, 1 gene (Unigene12273) encoding WRKY TF was downregulated. Previous studies revealed that *OsWRKY13* mediates rice disease resistance by regulating defense-related genes in the SA- and JA-dependent signaling pathways [46], and *Arabidopsis WRKY62* negatively regulates JA-responsive gene expression [47]. The down-regulation of *LOX* (Unigene22463) in the *P. coffeae*-challenged resistant ramie cultivar was consistent with that of the WRKY gene (Unigene12273), indicating that this LOX isoform may be positively regulated by the ramie WRKY encoded by Unigene12273. Many plant ERFs act as positive or negative regulators of JA-mediated resistance [44,48,49,50]. After the *P. coffeae* inoculation of resistant ramie, 3 ERF genes exhibited upregulated expression and showed coincident expression with that of defense proteins; thus, they may positively regulate JA-mediated defense responses.

## 4. Experimental Section

All experiments were carried out in a greenhouse and laboratory of the Institute of Bast Fiber Crops, Chinese Academy of Agricultural Sciences (IBFC, CAAS) in Changsha, China, with the exception of the preparation and sequencing of the transcriptome and digital expression gene libraries.

### 4.1. Plants and Nematode Inoculation

Ramie cultivars Qingdaye and Heipidou, resistant (disease index 10.4) and susceptible (disease index 82.9) to the root lesion nematode [51], respectively, were used in this study. Only the former was used for transcriptome and digital gene expression (DGE) analysis. Both cultivars were used for the comparison of the differences in gene expression between incompatible and compatible plants via quantitative real-time PCR (qRT-PCR) analysis. Ramie seedlings were prepared via cutting propagation by method described as following, in a greenhouse of IBFC, CAAS. The stem shoots were removed for promoting generation of lateral branches when ramie plants grow up to about 50 cm. Shoot tips of lateral branches extended to 10–15 cm in length were picked, and removed most leaves except for 2–4 leaves on the top with a sharp blade. After surface sterilization for 5 min by soaking in potassium hypermanganate (*w*/*v*, 0.08%) solution, each shoot tip was placed in a 250 mL triangular flask with 200 mL autoclaved tap water for culturing seedling at room set temperature ranged from 25 to 28 °C. When the roots of shoot tip extended to about 10 cm in length, seedlings were transplanted in a plastic pot (9 cm in diameter and 10 cm in depth) with nutritious soils (clay:sand:organic matter = 3:1:1, *v*/*v*) and cultured at room temperature. The soil was autoclaved and allowed to cool down at room temperate before transplant.

*P. coffeae* isolation YJ1100 [52], maintained on carrot discs, was used as the inoculum. To prepare nematode challenged (CH) and unchallenged control plants (CK), 1 mL water with or without 150 nematodes (mixture of juveniles and adults) was added directly to the soil near the roots of each ramie seedling. Three independent biological replications were performed for both the inoculated and control plants. One, 3 and 5 days post inoculation (dpi), both the challenged and unchallenged plants were uprooted, after which the roots of each plant were washed with tap water and blotted dry. Then, the clean roots were immediately frozen in liquid nitrogen and stored at −80 °C prior to RNA extraction. To prepare samples for transcriptome and digital gene expression analysis, half of the roots of three individual CK or CH plants, sampled simultaneously, were equally pooled and considered to constitute a sample. The remaining half of each plant was not pooled, and used for gene expression analysis by qRT-PCR.

### 4.2. RNA Preparation

The total RNA of each sample was extracted using an EASYspin plus Total RNA kit (Aidlab, Beijing, China), following the manufacturer’s protocol. The integrity and yield of the RNA were determined using an Agilent 2100 Bioanalyzer (Agilent Technologies, Palo Alto, CA, USA) and a Nanodrop 2000 spectrophotometer (Thermo Fisher Scientific, Wilmington, DE, USA), respectively.

### 4.3. Transcriptome Library Preparation and Sequencing

Illumina sequencing was performed at Biomarker technologies Co., Ltd., Beijing, China. For transcriptome sequencing, the equally mixed RNA (5 μg) of CK samples collected at different time intervals was used. Briefly, mRNA was purified from mixed total RNA by binding the RNA to magnetic oligo (dT) beads, after which it was broken into short fragments by adding fragmentation buffer. The short mRNA fragments were then used as templates to synthesize the first-strand cDNA using a random hexamer-primer, after which dNTPs, RNase H, DNA polymerase I and GEX second strand buffer were added in order to synthesize the second-strand cDNA. The resulting cDNA fragments were purified using a QiaQuick PCR extraction kit (QIAGEN, Hilden, Germany), and subsequently eluted in EB buffer for end repair and poly (A) addition. After being selected via agarose gel electrophoresis, suitable fragments were recovered and used as templates for PCR amplification. The amplification product yielded a cDNA library with inserts approximately 200 bp in size. Finally, the amplified library was sequenced using the lllumina HiSeq™ 2000 platform (Illumina, San Diego, CA, USA), and both ends of the inserts were sequenced. The 90 bp raw pair end (PE) reads were generated by the Illumina Genome Analyzer II system (Illumina, San Diego, CA, USA).

### 4.4. De Novo Assembly and Functional Annotation

The clean reads were obtained from the data by filtering out adaptor-only and low-quality reads (shorter than 13 bp, or reads containing more than 5% unknown nucleotides). *De novo* assembly of the clean reads was then carried out in order to generate non-redundant unigenes using the Trinity method [53], with an optimized K-mer length of 25. Clean read data have been submitted to the sequence read archive in NCBI, with the Sequence Read Archive accession number SRR1176934.

The unigenes were first searched against the NCBI Nr, Swiss-Prot, COG, and KEGG protein database using local BLASTx tool (with *E* value < 10^−5^) to obtain protein function information. With Nr annotation, we used the Blast2GO program (https://www.blast2go.com/) to acquire GO annotations according to their molecular functions, biological processes, and cellular component ontology [54]. Then, the WEGO software (http://wego.genomics.org.cn/cgi-bin/wego/index.pl) was used to perform the GO functional classification of all unigenes [55]. The sequences of the unigenes were also aligned to the COG database, in order to predict and classify possible functions. Pathway assignments were made by mapping the unigenes to the reference canonical pathways in the KEGG.

### 4.5. Digital Gene Expression (DGE) Library Preparation and Sequencing

To compare the expression of genes in ramie roots before and after *P. coffeae* attack, the DGE libraries of CK and CH samples were constructed using the method for transciptome analysis, as described above, though only single ends of the inserts were sequenced. The total RNA (5 μg) of the CK and CH samples were used, respectively, which were prepared by mixing equal amounts of RNA from plants sampled at three time points.

### 4.6. Identification of Differentially Expressed Genes (DEGs)

Clean reads from the CK and CH libraries were mapped to reference sequences in the transcriptome library above, using Blat software (http://genome.ucsc.edu/) [56]. Gene expression levels were measured during the RNA-Seq analysis (Biomarker technologies Co., Ltd., Beijing, China) as reads per kilobase of exon model per million mapped reads (RPKM) [57]. The DEseq software program (http://www-huber.embl.de/users/anders/DESeq/) was used to identify DEGs in a pairwise comparison, and the results of all statistical tests were corrected for multiple testing via the Benjamini–Hochberg false discovery rate (FDR < 0.01) [58]. If the adjusted *p* value obtained via this method was <0.001, and there was at least a twofold change (>1 or <−1 in the log_2_(ratio) values, which were calculated using the average RPKM value between the CK and CH DGE libraries), the gene was deemed to be significantly differentially expressed. The data from the DGE analysis (clean reads, counts, and RPKM values) have been submitted to the Gene Expression Omnibus in NCBI, with the GEO accession number GSE64974. To detect transcripts of nematode in CH library, reference sequences (56,325 contigs and singletons, kindly supplied by Godelieve Gheysen from the department of molecular biology, Ghent University, Ghent, Belgrium) in the transciptome of *P. coffeae* [18] were used. Reads in CH library were firstly mapped to reference sequences of ramie; then the unmapped reads were mapped to reference sequences of *P. coffeae*. Sequences of DEGs obtained were compared with reference sequences of *P. coffeae* in a local database using Blastn (with *E* value < 10^−5^) to check if any of them were from nematode. The local *P. coffeae* sequences database was constructed using NCBI-blast (version 2.2.31) software (ftp://ftp.ncbi.nlm.nih.gov/blast/executables/blast+/LATEST/).

### 4.7. Quantitative Real-Time PCR

To confirm that genes were differentially expressed in challenged and unchallenged samples, qRT-PCR was performed using the iQ SYBR Green Super Mix kit (Bio-Rad, Hercules, CA, USA) on an iCycler iQ system (Bio-Rad). First-strand cDNAs were synthesized from 1 μg of the total RNA of the CK or CH root samples collected at different time points, using a RevertAid First Strand cDNA Synthesis Kit (Thermo Scientific, Fermentas, Vilnius, Lithuania). The gene-specific primers for 15 candidate genes (Table S4) were designed using the Primer Premier 5.0 software program (Premier Biosoft, Palo Alto, CA, USA). Ramie *Actin1* gene (DQ665832) was used as the endogenous control. Each qRT-PCR reaction contained 2.0 μL of cDNA template from the reverse-transcribed reaction, 10 nM gene-specific primers, 10 μL of iTaq™ Universal SYBR Green supermix (Bio-Rad) in a final volume of 20 μL. Amplication was performed using the following programs: 95 °C for 30 s, followed by 40 cycles of 95 °C for 10 s, and 55 °C for 25 s. All reactions were performed in triplicate for the mixed samples, and in duplicate for the unmixed sample of each biological replicate. The expression data were transformed and analyzed using 2^−ΔΔ*C*t^ method [59]. After normalization with the expression level of the *Actin1* gene, the ratios were expressed as fold-changes compared to the expression levels detected in the control samples.

## 5. Conclusions

In this study, we reported first a comprehensive ramie root unigene pool (40,826 unigenes) generated by *de novo* assembly of RNA-Seq data. It is a valuable resource for studying molecular biology of ramie. Based on these data, we compared the transcriptome of *P. coffeae*-challenged and un-challenged ramie roots by digital gene expression analysis and 137 genes were identified to be significantly differentially expressed. Time-course expressions of eight defense-related genes in susceptible and resistant ramie cultivars were different after *P. coffeae* inoculation. The differential expression of protease inhibitors, pathogenesis-related proteins (PRs), and transcription factors in resistant and susceptible ramie during *P. coffeae* infection indicated that cystatin likely plays an important role in nematode resistance.

## Supplementary Materials

Supplementary materials can be accessed at: http://www.mdpi.com/1422-0067/16/09/21989/s1.
