# Supplementary material for: Identification of Ramie Genes in Response to Pratylenchus coffeae Infection Challenge by Digital Gene Expression Analysis
Source: Int J Mol Sci. 2015 Sep 11;16(9):21989–2007. doi: 10.3390/ijms160921989 (PMC4613293; doi:10.3390/ijms160921989)
Supplement: Supplementary file 1 [file ijms-16-21989-s001.zip › ijms-90123-Supplementary Information/ijms-90123-Supplementary Information.pdf]

Supplementary Information

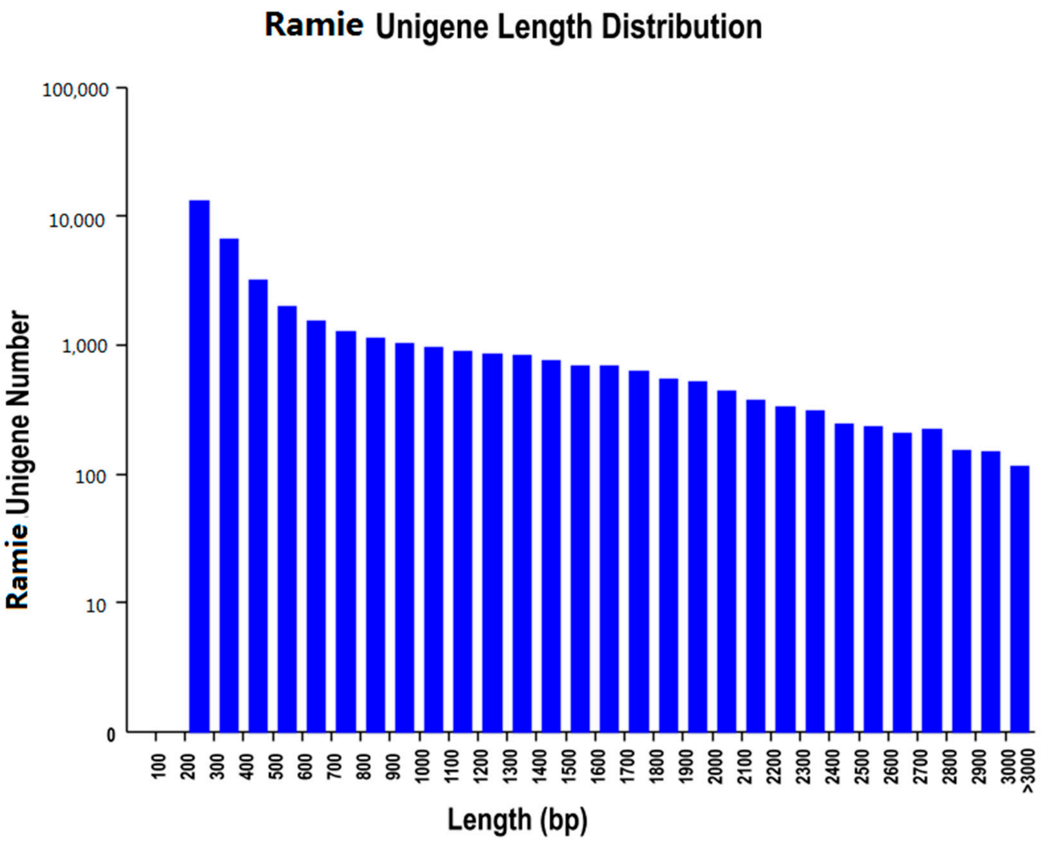

Figure S1. Length distribution profiles of unigenes in the cDNA library of fibrous ramie roots.

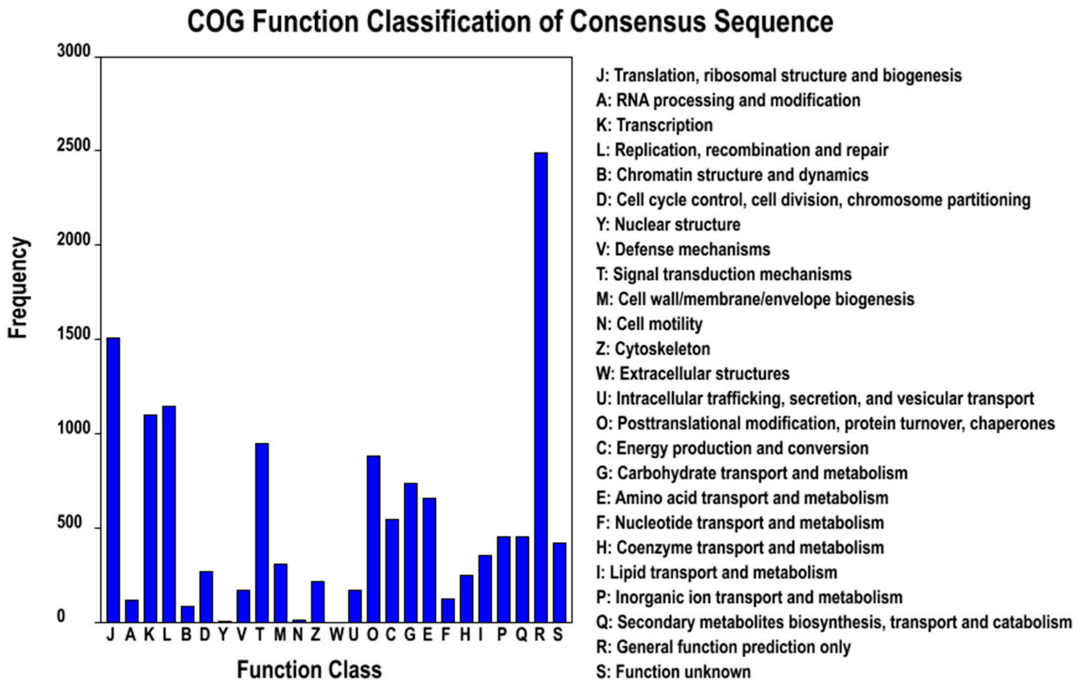

Figure S2. Unigenes distribution in COG groups based on classification. 9657 of total unigenes have COG classification within 25 categories.

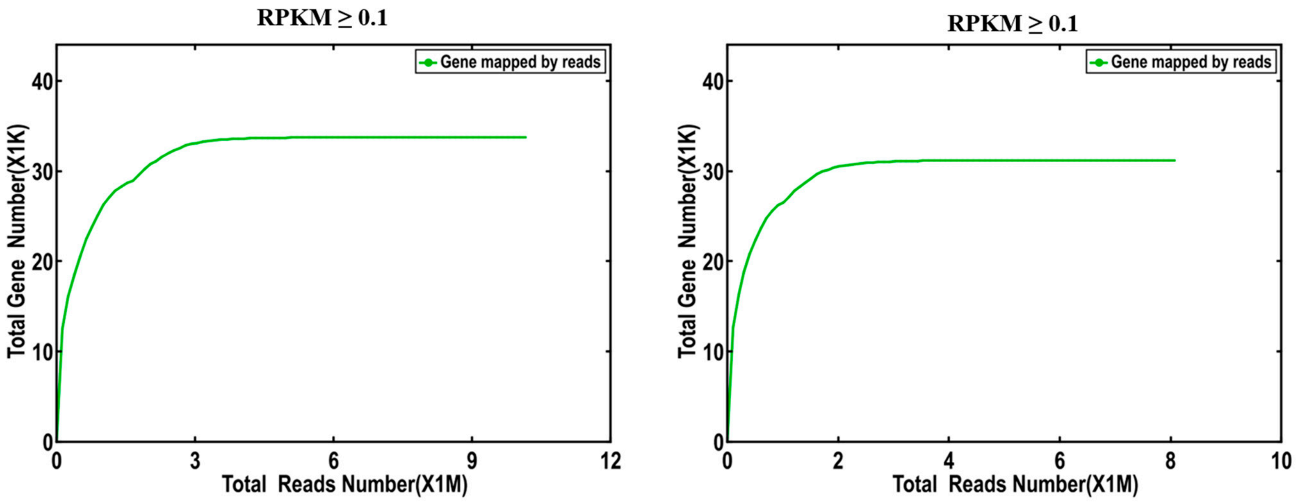

Figure S3. Saturation analyses of the CK (**left**) and CH (**right**) libraries.

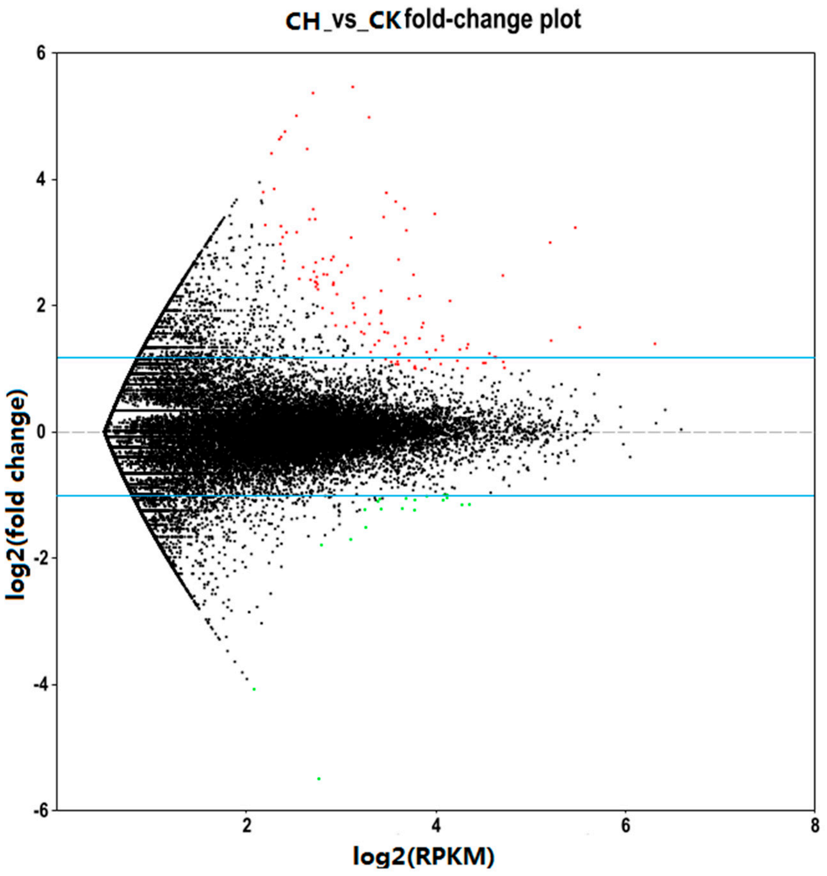

Figure S4. Comparison of gene expression levels between CK and CH libraries. Red and green dots represent transcripts more and less prevalent in the CH library, respectively, while black dots indicate transcripts that did not change significantly. The parameters "FDR $\leq$ 0.001" and "$\log_2$|Ratio| $\geq$ 1" were used as the threshold to judge the significance of gene expression differences. The blue lines above and below the zero line indicate 1 and −1, respectively.

Table S1. KEGG classification of ramie genes involved in defense pathways.

Pathway	Pathway ID	Gene Number
ABC transporters	ko02010	9
Basal transcription factors	ko03022	35
Endocytosis	ko04144	96
Peroxisome	ko04146	71
Phagosome	ko04145	115
Plant hormone signal transduction	ko04075	158
Plant-pathogen interaction	ko04626	132
Proteasome	ko03050	58
Regulation of autophagy	ko04140	22
SNARE interactions in vesicular transport	ko04130	22
Spliceosome	ko03040	156
Ubiquitin mediated proteolysis	ko04120	95

Table S2. Statistics of mapping DGE reads to reference transcriptome database.

Sample	CK	Percent	CH	Percent
Total Reads	10,155,914	100%	8,070,704	100%
Mapped Reads	8,207,281	80.81%	6,490,444	80.42%
Perfect Mapped Reads	4,901,290	59.72%	3,766,310	58.03%
Mismatch Reads	2,972,932	36.22%	2,424,148	37.35%
Indel Reads	164,150	2.00%	142,049	2.19%
Indel and Mismatch Reads	168,909	2.06%	157,937	2.43%
Identities	99.27%	–	99.24%	–

Table S4. Primer sequences used in qRT-PCR.

Genes	Forward 5'–3'	Reverse 5'–3'	Description
Unigene5469	GTGCCAAGGCGAGTCAAG	GCTCCAAGAGTCCGAAGG	Ser/Thr-rich protein T10
Unigene9043	GAAGGTTCCGAGCCAGAC	TTCGTTCGCATAGAAGAGTT	Ethylene-responsive transcription factor
Unigene9323	GTGGGAACAAATGGAAAT	TAGCCAACTGTAGGAACT	Proteinase inhibitor
Unigene2183	GGTTGCTGCTATTGATGTG	GGATGATTGTCGCCCTTA	Vegetative cell wall protein gp1
Unigene3206	GCAAGAGCATCAACCCAGAC	CGTGTTCCTCGGAATTAGC	Heat shock protein 70 kd
Unigene13732	TTCCCGCCCAGGTCCTTCCA	TCACCATAATGTACCGCCAAA	Polyphenoloxidase
Unigene19135	AGCCAAGGACATTAACAA	CGGAAGAATACGGCGAGA	Trypsin inhibitor
Unigene24094	GTAGTCGCAGTTTCCTCC	GAATGTCGGTAACCCTCC	Chitinase
Unigene9085	ACAAGCCACCACCAACCC	ACTATTGGCGGAAGCGTA	Proline-rich cell wall protein
Unigene7762	TATGACCTCCGCCGTTGA	CCACCACCAGAAGCACCA	Superoxide dismutase
Unigene11292	CACGGCGATTTAGACATA	GTGAAGGCGAAACAGCAA	Cysteine proteinase inhibitor
Unigene21351	GTTGATGATGGCTGGGTT	CGACATGGCTCTGTTCTT	Scarecrow-like protein 13-like
Unigene13343	TCTGTGGTCAATGGAGGCG	GGAGAACTTTGCGGTGGG	Non-specific lipid-transfer protein-like protein
Unigene22463	GAGGCGGATCGAGAAGGA	GCTCATAAGGCAACACCC	Lipoxygenase
Unigene6416	TGCGGGTCCCAGTGTCCTT	CTTGCCGTTGGTTCGTCT	Nod26-like protein
Actin1	AGTGGAGAAGCGTGGTTT	ATGAATGACGATAATGGC	Actin 1
